# Mobile health and hypertension in the digital era: patient perspectives on mHealth adoption and self-management in Saudi Arabia aligned with the digital health objectives of vision 2030

**DOI:** 10.2478/abm-2025-0031

**Published:** 2025-10-31

**Authors:** Sultan M. Alshahrani

**Affiliations:** Department of Clinical Pharmacy, College of Pharmacy, King Khalid University, Alfaraa, Abha 62223, Saudi Arabia

**Keywords:** digital health, health reformation, hypertension, mHealth

## Abstract

**Background:**

Hypertension remains a prominent public health concern in Saudi Arabia, contributing to cardiovascular morbidity and mortality. With widespread smartphone use and the national push toward digital transformation under Saudi Vision 2030, mobile health (mHealth) applications are a promising option for increasing self-management among hypertension patients.

**Objective:**

This study aims to analyze the acceptance, preferences, and perceived barriers toward utilizing mHealth applications for hypertension self-management among patients in Saudi Arabia.

**Methods:**

A cross-sectional survey was conducted from January to March 2025 utilizing an online, self-administered questionnaire disseminated across the 5 administrative regions of Saudi Arabia. The study investigated demographics, clinical factors, technology utilization, preferred app features, attitudes, and readiness to adopt mHealth solutions. Descriptive statistics and Pearson correlation analysis were employed.

**Results:**

Among the 1098 respondents, 55.3% were female, and the mean age was 45.0 ± 11.8 years. A majority (95.2%) owned cellphones, although only 22.1% had used a health-related app, and 11.8% had used one for hypertension. Most participants reported great interest in app features like medication reminders (78.4%) and blood pressure tracking (71.9%). Willingness to utilize mHealth was high (85.6%), and substantially linked with education level (*P* = 0.008) and number of drugs (*P* = 0.03).

**Conclusion:**

The study reveals considerable support for mHealth integration into hypertension care in Saudi Arabia. Tailoring app design to user demands and aligning deployment with Vision 2030’s digital health agenda can boost chronic illness management and patient empowerment nationally.

Hypertension is an important public health concern globally and is increasingly prevalent in Saudi Arabia, contributing to cardiovascular morbidity and mortality. National health reforms under Saudi Vision 2030 aim to reduce such pressures through digital innovation and patient-centered care paradigms [1, 2]. Despite the availability of antihypertensive therapies, control rates remain inadequate due to factors such as poor adherence, lack of follow-up, and limited patient engagement [3, 4].

Mobile health (mHealth) applications offer a promising strategy to enhance self-management among patients with chronic illnesses, particularly hypertension [5, 6]. In Saudi Arabia, the opportunity is facilitated by extensive smartphone ownership, topping 90% of the population [7]. Government-led platforms, such as Seha and Sehaty, have previously demonstrated better access to and continuity of care, particularly during the COVID-19 pandemic [8, 9]. However, disease-specific applications targeting hypertension remain underutilized and understudied in the region.

A study conducted by Jiménez-Zarco et al. [10] shows that throughout the world, significant variations occurred in mHealth service acceptance during the COVID-19 pandemic. Teleconsultation, approach to patient information, and assignment reminders were the most enforced services, highlighting the importance of distant care during health crises.

International data significantly support the effectiveness of mHealth in hypertension control. A systematic review indicated that 71.4% of mobile-based interventions effectively decreased blood pressure (BP), whereas randomized controlled trials demonstrated increased medication adherence and lifestyle change [5, 6, 11]. However, the success of such tools depends enormously on usability, relevance, and integration with the local healthcare system [12, 13].

In the Saudi context, Alzahrani et al. [14] emphasized the necessity for culturally appropriate, Arabic-language apps. Similarly, studies have demonstrated that user confidence and perceived usefulness are crucial for adoption [15, 16]. Factors such as age, gender, digital competence, and the burden of comorbidities influence engagement with mHealth tools [17, 18]. Notably, men and urban people in Saudi Arabia demonstrate stronger digital preparedness than their rural or older counterparts [19, 20].

Digital transformation activities under Saudi Vision 2030 specifically advocate for the promotion of telemedicine and eHealth to improve efficiency and patient satisfaction [1]. The success of national platforms like Seha indicates that patients are welcoming digital solutions, particularly when they are endorsed by public health authorities and linked to existing care systems [8, 21]. Digital health technologies, such as mobile phone applications, wireless devices, and electronic health record systems, have significantly increased global health security [22]. The rapid evolution of mobile health applications (mHealth apps) has become increasingly important in enhancing healthcare delivery, especially during the COVID-19 pandemic worldwide, including Saudi Arabia [23].

Despite the positive outlook, obstacles persist. Studies show that concerns regarding privacy, low awareness, and a lack of customization hinder adoption [24, 25].

Our study intends to address these gaps by examining the attitudes, preferences, and perceived barriers of hypertension patients in Saudi Arabia regarding mHealth solutions. By focusing exclusively on this population and linking our work with Saudi Vision 2030 targets, we give vital insights into building effective, scalable, and inclusive digital health treatments.

## Methods

A descriptive, cross-sectional study was performed between January and March 2025 to evaluate acceptability and attitudes regarding mHealth applications for hypertension self-management among adult patients in Saudi Arabia. The study was conducted across all 5 administrative regions of the Kingdom (Central, Eastern, Western, Northern, and Southern) via an online survey.

### Study population

Eligible participants included those who were diagnosed with hypertension, aged 18 years or older, residents in Saudi Arabia, and able to read and speak Arabic. Exclusion criteria included people diagnosed with secondary hypertension or cognitive problems that could restrict the capacity to respond independently, or who were unwilling to answer the survey. Participation was completely voluntary and anonymous.

A convenience sample approach was employed to distribute the questionnaire electronically via popular social media platforms (WhatsApp, X [previously Twitter], Telegram). Based on a confidence level of 95%, an anticipated population proportion of 50%, and a 3% margin of error, the minimum sample size was calculated to be 1067 participants using the Raosoft sample size calculator. Ultimately, 1098 complete replies were obtained and included in the final study.

### Survey instrument

The data collection tool was a structured, self-administered questionnaire designed in Arabic after a thorough review of the literature and adaptation of validated approaches from a similar research project [5]. The questionnaire consisted of 5 sections:

Demographics and clinical characteristics: age, gender, marital status, education, employment status, region of residence, years from hypertension diagnosis, number of antihypertensive drugs, and presence of comorbidities. Technology access and usage: ownership of cellphones, internet usage, familiarity with health-related apps, and sources of health information. Preferred app features: participants identified preferred features of a hypertension self-management app, including medication reminders, BP tracking, food logs, physical activity monitoring, stress management, and communication with healthcare specialists. Attitudes toward mHealth apps: This component includes 9 statements scored on a 5-point Likert scale (1 = Strongly Disagree to 5 = Strongly Agree) modified from past studies [3, 5, 6] measuring perceived ease of use, benefits, and barriers. Willingness and Support: Questions assessed willingness to use mHealth apps, confidence in use, and the need for help from family or friends.

The test was piloted on 15 hypertensive patients for clarity and reliability. Minor linguistic improvements were made based on feedback. The Cronbach’s alpha for the attitude scale was 0.86, showing strong internal consistency.

### Data collection

Data were collected using Google Forms. Participants provided implied consent before beginning the survey. Confidentiality and anonymity were strictly maintained.

### Data analysis

Data were transformed to Microsoft Excel and analyzed using IBM SPSS version 26.0. Descriptive statistics (frequencies, percentages, mean ± standard deviation) were employed for all variables. Inferential methods, including chi-square testing and Pearson correlation, were utilized to evaluate associations between demographic/clinical characteristics and willingness to utilize mHealth apps. A *P* value of 5% (*P* < 0.05) was considered statistically significant.

## Results

### Participant characteristics

A total of 1098 individuals who have hypertension participated in this study. As shown in Table 1, the majority were female (55.3%) and married (65.2%), with a mean age of 45.0 ± 11.8 years. Most respondents were employed (60.2%) and resided in metropolitan areas (71.1%). In terms of education, 35.7% possessed a university degree, while 20.2% had finished a diploma. A considerable number of the individuals (55.6%) reported having at least one comorbid condition, such as diabetes or cardiovascular disease. Nearly half (45.3%) reported using two hypertensive medications daily, and over one-third (35.4%) had been diagnosed with hypertension for more than 6 years.

**Table 1.** Demographics and clinical characteristics

Characteristic	N (%)
Gender	
Male	491 (44.7)
Female	607 (55.3)
Mean age (years) 45.0 ± 11.8	
Marital status	
Married	716 (65.2)
Single	275 (25)
Divorced/widowed	107 (9.8)
Education	
Primary	98 (8.9)
Middle/high school	183 (16.7)
Diploma	222 (20.2)
University	392 (35.7)
Postgraduate	203 (18.5)
Employment status	
Employed	661 (60.2)
Unemployed	274 (25)
Retired	163 (14.8)
Residence	
Urban	781 (71.1)
Rural	317 (28.9)
Years with HTN	
<1	110 (10)
1–3	276 (25.1)
4–6	323 (29.4)
>6	389 (35.4)
Daily meds	
1	242 (22)
2	497 (45.3)
3+	277 (25.2)
Comorbidities	
Yes	610 (55.6)
No	488 (44.4)

### Technology access and use

As seen in Table 2, the majority of respondents (95.2%) owned a smartphone, and 89.7% reported using the internet to search for health-related information. However, only 22.1% had previously used a health-related mobile application, and just 11.8% had experience with an app specifically designed for hypertension management. Physicians were the most common source of hypertension-related information (61.4%), followed by internet searches (18.9%) and input from family and friends (13.2%).

**Table 2.** Technology use and information sources

Technology usage	N (%)
Owns smartphone	1046 (95.2)
Uses internet for health info	985 (89.7)
Used any health app	243 (22.1)
Used hypertension app	130 (11.8)
Source of health info	
Physician	674 (61.4)
Family	145 (13.2)
Social media	71 (6.5)

### Preferred features in a hypertension mHealth app

Participants were asked to indicate the features they would find most useful in a mobile app to manage their hypertension. As shown in Table 3, the most frequently desired feature was medication reminders (78.4%), followed by BP tracking and history (71.9%) and diet/sodium intake monitoring (64.5%). Other preferred features included physical activity tracking (60.8%), stress management tools (47.3%), and communication capabilities with healthcare providers (42.6%). Less frequently selected but still notable were alerts for abnormal BP readings (39.1%) and peer support functionality (21.5%).

**Table 3.** Preferred mHealth app features

Preferred feature	N (%)
Medication reminders	860 (78.4)
BP tracking	790 (71.9)
Diet/sodium monitoring	709 (64.5)
Physical activity tracking	668 (60.8)
Stress management	520 (47.3)
Communication with providers	468 (42.6)
BP alerts	429 (39.1)
Peer support	236 (21.5)

1 BP, blood pressure.

### Attitudes toward mHealth applications

Attitudes toward the use of mHealth applications were assessed using a 5-point Likert scale across several statements. As detailed in Table 4, the majority of participants expressed positive attitudes. Specifically, 94.1% agreed or strongly agreed that they would use an app if it were free, and 92.6% agreed they would try one if it were easy to operate. A similar proportion (90.4%) indicated that reminder functions would help them follow medical advice, while 91.8% felt that apps could assist in monitoring BP effectively. The mean scores for these statements ranged from 4.18 to 4.39 out of 5, with standard deviations indicating relatively low variability across responses. Despite the overall optimism, 8.5% of respondents expressed privacy concerns, selecting neutral or negative responses on that item.

**Table 4.** Attitudes toward mHealth apps

Statement	Strongly agree n (%)	Agree n (%)	Neutral n (%)	Disagree n (%)	Strongly disagree n (%)	Mean	SD
Would use if free	528 (48.1)	505 (46.0)	34 (3.1)	21 (2.0)	8 (0.8)	4.39	0.72
Would use if easy to use	494 (45.0)	522 (47.6)	55 (5.1)	19 (1.8)	5 (0.5)	4.35	0.7
Would reduce clinic visits and costs	423 (38.6)	533 (48.6)	81 (7.4)	37 (3.4)	21 (2.0)	4.18	0.86
Reminders to follow advice	490 (44.7)	501 (45.7)	64 (5.9)	34 (3.1)	6 (0.6)	4.31	0.77
Helps monitor BP	507 (46.2)	500 (45.6)	55 (5.1)	21 (2.0)	12 (1.1)	4.34	0.76
Facilitates communication	465 (42.3)	509 (46.3)	77 (7.0)	30 (2.7)	17 (1.7)	4.28	0.79
Privacy protected	419 (38.1)	524 (47.7)	93 (8.5)	45 (4.1)	17 (1.6)	4.23	0.81
Willing to use mHealth	490 (44.6)	450 (41.0)	110 (10.0)	33 (3.0)	15 (1.4)	4.24	0.82
Confident to use independently	393 (35.8)	411 (37.4)	156 (14.2)	90 (8.2)	48 (4.4)	3.91	1.02
Needs family help	289 (26.3)	397 (36.2)	231 (21.0)	115 (10.5)	66 (6.0)	3.6	1.14

1 BP, blood pressure.

Additionally, 85.6% of participants expressed willingness to use a mobile app for hypertension self-management, with 73.2% confident in their ability to use it independently. Among those who were less confident, 62.5% stated they would seek help from a family member to operate the app.

Furthermore, to explore associations between demographic and clinical characteristics and willingness to use mHealth apps, correlation analyses were conducted (Table 5). A statistically significant positive correlation was found between willingness and both education level (*P* = 0.008) and the number of daily antihypertensive medications (*P* = 0.03). No significant association was found with age (*P* = 0.11) or duration since hypertension diagnosis (*P* = 0.09).

**Table 5.** Correlation between characteristics and willingness to use mHealth apps

Variable	Correlation coefficient	*P*
Age (years)	–0.042	0.11
Education level	0.198	0.008*
Years since diagnosis	0.092	0.09
Number of medications	0.137	0.03*

* Statistically significant *P* = 0.05.

## Discussion

The current study highlights substantial acceptance among Saudi patients with hypertension for mHealth applications, with 85.6% indicating willingness to use an app and 95.2% possessing smartphones. These findings are consistent with international studies that demonstrate great practicality and patient acceptance of digital tools in chronic illness management [5, 6, 11]. However, this study is among the first in Saudi Arabia to focus completely on hypertension, a condition commonly overshadowed by studies on diabetes and general chronic care [3, 4, 20].

Participants prioritized medication reminders, BP monitoring, and access to physicians—features shown in previous studies to promote adherence and empower patients [14, 15, 25]. Over 73% of our respondents felt secure using an app independently, showing a significant shift in digital health literacy, especially among educated users.

This study’s inferential analysis found that readiness to utilize mHealth applications was substantially connected with education level and the number of daily drugs. No significant association was detected with age or duration of diagnosis. These findings emphasize the need to customize interventions toward patients with complex treatment regimens who already see the value in technological support.

This coincides with Saudi Vision 2030 goals to use digital solutions that improve access, empower patients, and eliminate unnecessary clinic visits [1]. National apps like Seha and Sehaty have demonstrated this paradigm by boosting service delivery and user satisfaction, particularly during the COVID-19 era [8, 9]. Our study expands on this foundation by determining user-specific preferences and readiness for disease-specific mHealth integration.

Barriers such as concerns about privacy and usability were similar to prior reports, both worldwide and within the Kingdom [21, 22]. Ezenwaji et al. observed that low security awareness can limit adoption [21]. Similarly, worldwide studies suggest that usability and trust must be considered early in app development [11, 26]. These findings underline the need for co-designing digital tools with users, implementing simple interfaces, and delivering training for vulnerable populations.

Comparatively, studies from China, Germany, and the UK support the current study’s conclusions, but also demonstrate that Western users may rely less on provider participation [6, 25, 27–30]. In Saudi Arabia, the physician-patient interaction remains fundamental to care, suggesting that mHealth tools should operate as adjuncts rather than substitutes.

Gender and urban-rural variations in digital participation have been previously identified [17, 18]. This emphasizes the necessity for inclusive rollout tactics that incorporate socioeconomic and educational inequalities. Additionally, usability assessment, often disregarded, is vital for guaranteeing continuous engagement, as stressed by Sousa et al. [12].

Unlike prior studies that broadly explore digital readiness, our work specifically examines hypertensive patients and offers real-world guidance on implementation. The strong user interest in features like appointment scheduling, remote monitoring, and lifestyle tracking confirms that such tools are well aligned with Saudi Vision 2030’s focus on preventive care and population health management [1].

## Conclusion

This study provides compelling evidence for the high acceptability and potential advantages of mHealth applications in the self-management of hypertension among patients in Saudi Arabia. The robust willingness to adopt such tools, especially among educated individuals and those managing complex drug regimens, demonstrates a favorable trend in digital health readiness across the Kingdom. The outcomes from this study demonstrate a high preference for user-centric features such as medication reminders, BP tracking, and communication with healthcare providers. Such decisions underline the significance of adding practical, intuitive, and culturally relevant capabilities to app development. Additionally, the statistically significant connections between mHealth adoption and important demographic characteristics underline the need for focused education and support initiatives to maximize reach and usage.

Importantly, this research complements and supports the goals of Saudi Vision 2030, which prioritizes healthcare transformation through digital innovation, patient empowerment, and greater telemedicine access. National apps like Seha and Sehaty have already proved the feasibility of digital integration. This study expands on this momentum by stressing the readiness of hypertension patients to embrace individualized, app-based therapies. By linking mHealth deployment with Saudi Vision 2030’s health sector transformation goals, Saudi Arabia can lead in defining a digitally enabled, patient-driven paradigm of chronic illness care. Future work should focus on usability, privacy, and system integration to enable sustainable uptake and measurable clinical outcomes.
